# Microarchitecture of *Python regius* Scale Surface: A Natural Strategy for Bacterial Adhesion Prevention

**DOI:** 10.1021/acsomega.5c12739

**Published:** 2026-03-11

**Authors:** Vaclav Peroutka, Katerina Navratilova, Vera Jencova, Jana Jiresova, Jana Mullerova, Simona Lencova

**Affiliations:** † Department of Biochemistry and Microbiology, 52735 University of Chemistry and Technology, Prague 166 28, Czech Republic; ‡ Department of Chemistry, Faculty of Science, Humanities and Education, 48261 Technical University of Liberec, Liberec 461 17, Czech Republic; § Department of Physics and Measurements, University of Chemistry and Technology, Prague 166 28, Czech Republic

## Abstract

Microscale surface structures on natural materials can provide unique functional properties, *inter alia*, for biological defense. Here, we report that the dorsal scales of ball python (*Python regius*), feature regularly distributed sharp microprotrusions (spikes) that may serve as a model surface for topography-driven prevention of bacterial adhesion and biofilm formation. The chemical composition and microarchitecture of the skin grain and flesh sides were characterized by Fourier Transform Infrared (FTIR) spectroscopy and scanning electron microscopy (SEM), confirming a keratin-rich, highly organized outer surface bearing dense arrays of spikes with micrometer-scale height and spacing. SEM imaging further corroborated markedly reduced colonization of the spike-bearing dorsal scale surface. Quantitative biofilm assays based on standard colony-forming unit (CFU) enumeration were performed using the newly developed scale-pair model. Relative to the smooth polystyrene reference, *Escherichia coli* and *Staphylococcus aureus* attachment and subsequent biofilm formation decreased by 88 and 78%, respectively, after 48 h of incubation in 37 °C. Other cultivation experiments ruled out chemical effects of any residual antimicrobial substances on the skin on bacterial growth, demonstrating that the topography alone mediates inhibition. These findings indicate that *P. regius* scale microstructures may function as a passive antimicrobial defense, and could inspire biomimetic, antibiofilm materials for biomedical and industrial applications.

## Graphical abstract

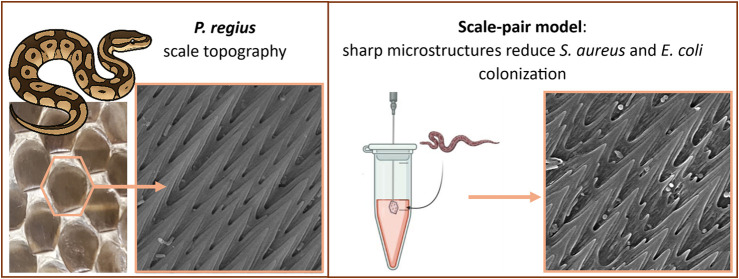

## Introduction

The challenge of bacterial biofilms has become one of the defining issues in contemporary microbiology, biomedical engineering, and industrial hygiene. Biofilms form when microbial cells adhere to surfaces, encase themselves in extracellular polymeric substances (EPS), and generate complex, resilient communities. The EPS matrix provides mechanical stability, traps nutrients, and impedes penetration of antimicrobial agents, making biofilm-associated microorganisms up to a thousand times more resistant to antibiotics and disinfectants than their planktonic counterparts.^1,2^ This resilience leads to persistent infections on medical devices, chronic wounds, and contamination of industrial equipment, imposing enormous costs on healthcare systems and industries worldwide. Conventional approaches relying on chemical antimicrobials are increasingly undermined by the rise of antimicrobial resistance.^3^ In addition, biofilm allows bacteria to control certain population processes through *quorum sensing* and greatly facilitates horizontal gene transfer—including antibiotic resistance genes, further exacerbating the problem of antimicrobial resistance.^4,5^ As such, there is an urgent need for nonchemical, sustainable strategies that prevent initial microbial adhesion and subsequent biofilm development.

Natural materials offer compelling inspiration for antifouling and antimicrobial surface design. Insects, marine organisms, and reptiles have evolved a rich variety of surface structures that endow them with unique functional properties. Insect wings, for example, carry nanoscale pillars that physically rupture bacterial cells upon contact.^6^ Shark skin, patterned with riblet-like denticles, reduces microbial colonization and drag simultaneously.^7^ The surfaces of lotus leaves and rose petals exhibit distinct hydrophobicity and adhesive properties driven by microscale papillae and nanostructured waxes.^8^ Among vertebrates, reptilian scales represent a fascinating but comparatively underexplored class of natural surfaces whose structural complexity may extend beyond locomotion and mechanical wear resistance into antimicrobial defense.

Recent research has demonstrated that micro- and nanoscale surface architecture can modulate bacterial attachment and biofilm formation, motivating the development of antibiotic-free, biomimetic antimicrobial surfaces.^9^ Depending on geometry and materials, topographic strategies may act primarily by reducing initial adhesion (antifouling/antiadhesive) or by contact-active effects that can damage cells mechanically;^10^ however, the reported efficacy and proposed mechanisms vary widely across studies and experimental conditions.^11^ Recent reviews highlight both the promise of topography-based approaches and the lack of consensus regarding which feature dimensions and arrangements are most effective, underscoring the need for well-defined model surfaces and careful characterization. In this context, naturally occurring structured biological interfaces provide valuable templates for linking feature geometry to bacterial responses.

Snake skin, respectively their scales, represents a particularly interesting model for studying structures and biological interactions. Shed specimens are ubiquitous in natural collections and provide a sustainable, ethically unproblematic source of material, obviating the need for direct experimentation on living animal models. Importantly, snake skin is also sufficiently durable to undergo treatments such as washing, irradiation, or high-pressure steam sterilization, procedures that effectively eliminate residual antimicrobial peptides, commensal skin microbiota, and immune-derived components that might otherwise confound analyses of biological interactions. Following such treatment, the shed skin is metabolically inert, chemically depleted of active antimicrobial molecules, and composed primarily of keratinized layers, ensuring that experimental outcomes predominantly reflect the influence of surface topography. These attributes make the scales from shed snake skin an optimal target for investigating the role of micro- and nanoscale topography in modulating microbial adhesion and biofilm development.

The surface of snake scales has been extensively studied in the context of friction anisotropy, locomotor performance, and optical properties.^12,13^ SEM and atomic force microscopy studies have revealed rich variation across species, scale positions, and orientations, from ridges and fibrils to sharp protrusions with submicron dimensions.^12,14^ Despite these advances in morphological characterization, the biological implications of such structures in preventing microbial colonization remain understudied. To address the broader question of how naturally occurring microtopographies can inform biomimetic antibiofilm design, we test whether the spike-bearing dorsal scales of *Python regius* suppress *E. coli* and *S. aureus* adhesion/biofilm formation via topography alone, using SEM-guided analysis, a scale-pair model, and a control excluding chemical interference of antimicrobial components of the skin. The presented study aims to address the existing research gap and provide inspiration for the development of biomimetic materials with antibiofilm properties.

## Materials and Methods

### Bacterial Species

In this study, two bacterial strains were used: Gram-positive *Staphylococcus aureus* ATCC 25923 (eq CCM 3953; origin: clinical isolate) and Gram-negative *Escherichia coli* ATCC 25922 (eq CCM 3954; origin: clinical isolate). The strains were obtained from the Czech Collection of Microorganisms (CCM, Czechia), stored in Tryptone Soy Broth (TSB, Oxoid Ltd., United Kingdom) mixed with 25% glycerol (Penta, Czechia) at −80 °C, and before each analysis, inoculated into sterile TSB (5 mL) and incubated for 24 h at 37 °C.

### Snake Skin Samples

Based on preliminary screening evaluating shed skins of several constrictor species, *Python regius* was selected as the primary model. The relatively large scales of *P. regius* facilitated reproducible handling and assembly of the scale-pair model for minimizing the effect of unstructured interscale regions of the skin. Moreover, the *P. regius* scales exhibited the most regular and uniform surface microtopography among the tested samples, enabling consistent analyses of topography-driven interactions. The snake skin sheds were donated by the Zoological and botanical garden Plzen, Czechia.

### Scanning Electron Microscopy

SEM was performed as described in our previous study.^15^ The snake skin and scale samples were attached to aluminum sample holders using double-sided carbon adhesive tape. In order to prevent surface charging, the samples were coated with a 10 nm layer of gold. The samples were observed using a Tescan Mira 3 LMH SEM (Tescan, Brno, Czech Republic) equipped with a Schottky cathode at an acceleration voltage of 15 kV. The lengths and mutual distances between equivalent sections of microstructures using 2D projection of SEM micrographs were calculated using MiraTC software.

### Infrared Spectroscopy (FTIR)

The chemical composition of the *P. regius* skin (grain vs flesh side) was analyzed by infrared spectroscopy using a Fourier transform spectrometer (FTIR) (Nicolet iZ10; Thermo Scientific, USA). The measurements were performed at room temperature, the samples were placed on an ATR diamond crystal for analysis, and the spectral analysis was performed in the infrared region in the range of 400–4000 cm^–1^ with a spectral resolution of 4 cm^–1^. We applied atmospheric and baseline corrections.

### Bacterial Growth in Snake Skin Presence – Verification of Chemical Interference

Snake skin samples were precooled for 15 min in liquid nitrogen and cryogenically ground to powder (196 °C; 20 repeats of 1 min grinding at 30 Hz and 2 min cooling), using CryoMill (Retsch GmbH, Germany). Resulting particle size was <50 μm. The powder was then suspended in sterile TSB (1.1 g/L), autoclaved, and distributed into 12- and 96-well plates at a final concentration of 1 g/L. Cultures were prepared with (i) bacterial strains at 0.5 McF or (ii) no bacteria (sterility control); bacteria in TSB served as growth references. Incubation was performed for 24 h at 37 °C. In 96-well plates, growth was monitored hourly with BioTek Synergy H1Multimode Reader (Agilent), and growth curves were generated.^16^

This cultivation experiment was performed as a control to exclude chemical interference from shed skin constituents. By sterilizing the shed skin the same way as in *the scale pair model experiment* and subsequently grinding it to powder prior to addition to the growth medium, we maximized the potential release of any residual bioactive compounds (e.g., antimicrobial peptides, lipids, or other small molecules). The absence of growth inhibition relative to the medium-only reference indicates that reductions in adhesion/biofilm growth observed in the scale-pair experiments arise purely from surface topography rather than leachable antimicrobial substances.

### Biofilm Formation assays

#### Crystal Violet Staining (Biofilm Formation Dynamics)

Biofilm formation was provided according to previously published protocol.^17^ 3 mL of bacterial suspension (TSB, 0.5 McF) were cultivated in a 12-well microtiter plate at 37 °C for up to 48 h. Mature biofilms were gently rinsed five times with sterile distilled water to remove nonadherent cells. The substrates were then air-dried for 45 min at room temperature in a laminar-flow box. For biomass visualization, biofilms were stained with 0.1% (w/v) crystal violet (CV; Sigma-Aldrich) for 45 min at room temperature. After staining, the samples were washed five times with sterile distilled water to eliminate excess dye. The resulting stained biofilms were documented by visual inspection and imaged using light microscopy at 60× magnification. Biofilms formed by bacteria cultivated in sterile TSB without snake skin powder were processed in parallel under identical conditions and used as the control.

### CFU Determination (Biofilm Formation on Snake Scale Samples)

Biofilm formation was provided as described in our previous study^18^ with a slight modification. As notel later in the Results and Discussion, a key methodological challenge is the spatial heterogeneity of shed skin: the sharp spike-like microprotrusions of interest are restricted to the outer face of selected dorsal scales, whereas interscale regions and other nonstructured areas can support conventional adhesion and thereby mask topography-dependent effects. To isolate the influence of scale microtopography in a manner relevant to biomimetic surface fabrication, we therefore developed a scale-pair model. Individual dorsal scales were cut from the snake skin bonded back-to-back using acetate silicone and then attached to a steel needle support so that only the scale surfaces were exposed to the bacterial suspension. This configuration maximized the area of spike-bearing topography while minimizing exposure of smooth, nonstructured regions (including interscale epidermis and the scale underside). The resulting assay preferentially reported adhesion and biofilm development governed by the dorsal scale microstructures.

Prepared assemblies were sterilized (rinsing in 70% ethanol, followed by 30 min UV–C exposure) immediately prior to use. Each model was immersed such that only the scale-bearing tip contacted the bacterial suspension (0.5 McFarland standard in TSB), thereby minimizing colonization of the metal support, and without the samples touching the walls of the tube. Incubations were performed statically at 37 °C for 48 h. A size-matched, smooth polystyrene object served as a reference control. CFU determination was performed according to published protocols.^15,17^ Briefly, biofilm-covered samples were rinsed five times with sterile 0.9% (w/v) NaCl in distilled water to remove loosely attached cells and then air-dried for 45 min at room temperature in a 12-well microtiter plate. Subsequently, 1 mL of sterile saline was added to each well, and the plates were sonicated for 3 min to detach and disperse biofilm-associated cells into suspension. The resulting suspensions were serially diluted 10-fold up to 10^–9^. Aliquots (20 μL) of each dilution were then plated as droplets onto TSA agar. Plates were incubated aerobically at 37 °C for 24 h, after which CFU were counted and expressed as CFU/cm^2^. In addition, biofilm formation was reported as a percentage relative to the reference substrate, polystyrene.

#### Controls

A polystyrene surface on a plate was used as a reference sample and treated similarly to the snake skin samples.

### Data Analysis

All experiments were performed in three independent biological replicates, each with three technical repeats per condition, to ensure robustness of the analysis. Quantitative data are presented as mean ± standard deviation (SD). Statistical significance was assessed using *t* test followed by Tukey’s post hoc test, with *p* < 0.05 considered statistically significant.

## Results and Discussion

Topography-driven antimicrobial design is an active area of research, with growing interest in natural templates that combine complex surface architectures with robust biological function. Reviews of the field emphasize that outcomes depend strongly on the type of response measured (e.g., inhibition of adhesion vs contact-active killing), the bacterial species, and how surfaces are characterized and tested. Our study contributes to this broader effort by examining a naturally occurring, highly regular spike-bearing dorsal scale microarchitecture and evaluating its influence on bacterial colonization using a test configuration designed to isolate the structured surface. By focusing on a reproducible biological microtopography and explicitly controlling for chemical interference, the work supports a primarily topography-mediated, antiadhesive/antibiofilm interpretation and informs biomimetic translation.^9–11^

Our investigation aimed to clarify whether snake scale topographies contribute to passive microbial defense by inhibiting bacterial adhesion and biofilm formation. We focused on the ball python (*Python regius*) scales, which topography has been studied in a few studies so far^12,19,20^ without targeting the sharp spikes for their antimicrobial relevance. SEM imaging (Figure 1, Figure 2) revealed the topographical differences in various parts of the skin sheds and their outer and inner sides, and notably the presence of sharp protrusions and ridge arrays on *P. regius* dorsal scales (Figure 1, Figure S1). The regularity of the sharp protrusions in terms of length and mutual distance between the spike tips and the lower part of the protrusions was measured and calculated on the 2D SEM micrographs using MiraTC software. The protrusion length reached up to 9.0 ± 0.7 μm, the distance of following tips was 4.6 ± 0.2 μm and the distance of adjacent tips was 4.4 ± 0.6 μm (for demonstration of those measurements see Figure S1).

**Figure 1:**
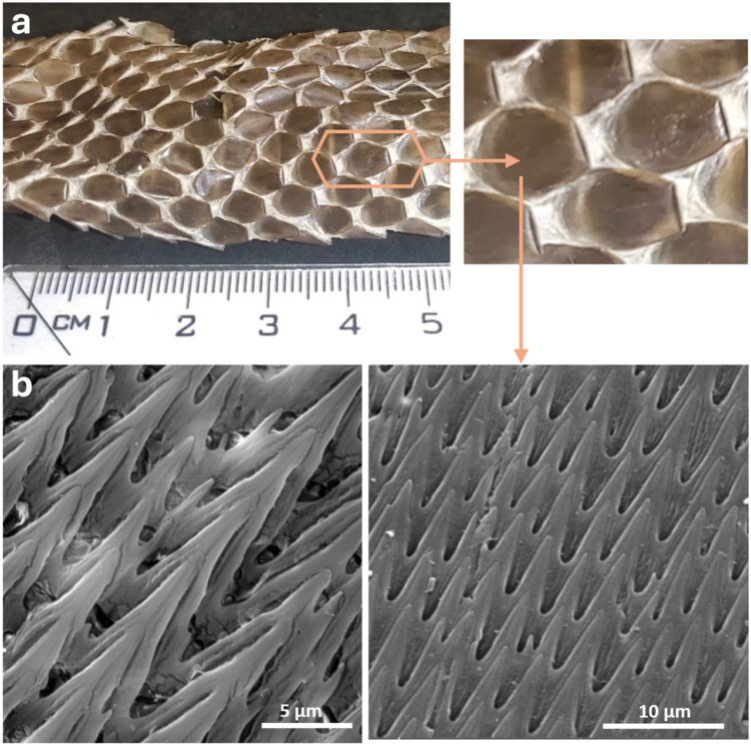
*P. regius* skin shed: (a) macroscopic view of target area with spikes, (b) sharp protrusions and ridge arrays on *P. regius* dorsal scales visualized using SEM.

**Figure 2:**
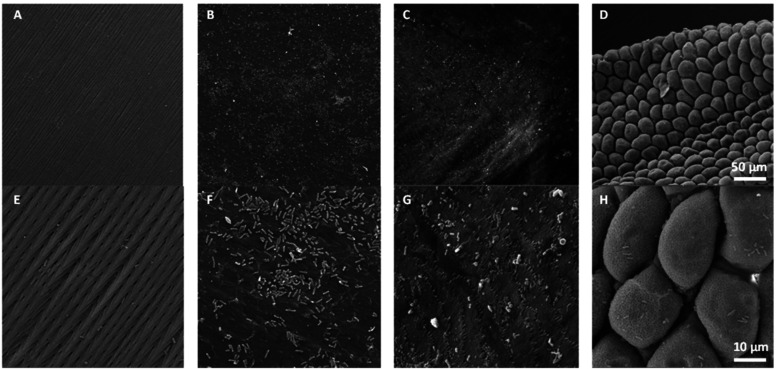
SEM analysis of *P. regius* skin, samples cocultivated with *E. coli*. The images demonstrate variability between different spaced on the skin: (A, E)—outer side of the scale with sharp protrusions, preventing bacterial adhesion; (B, F,C,G)—inner side of the skin without sharp protrusions, this absence enables bacteria to attach and colonize the skin; (D, H)—space between scales on the outer side of the skin, with relatively smooth structures enabling bacterial colonization.

The FTIR analysis (Figure S2) characterized the *P. regius* skin sample and confirmed the sample as animal skin. The grain side was identified as a keratin-rich, highly organized surface, while the flesh side was identified as a collagen-based connective tissue with a relatively higher lipid content. The analysis of the infrared spectrum confirms animal skin sample, showing a characteristic protein profile.^21^ The spectral data aligns well with published benchmarks for keratin and collagen tissues.^21,22^

We hypothesize that the regularly and densely distributed sharp protrusions could hinder bacterial attachment and colonization by preventing cell adhesion, microcolony formation, and bridging between structures—processes essential for mature biofilm development. To verify this, we have carried out biofilm formation assays on a sterile snake scale with two model organisms: *Escherichia coli* ATCC 25922 (Gram-negative) and *Staphylococcus aureus* ATCC 25923 (Gram-positive). These isolates were selected as model organisms because they represent common biofilm formers of clinical and industrial significance, and their distinct cell wall composition, surface charge, and adhesion strategies provide complementary perspectives for elucidating snake scale-bacteria interactions.

To eliminate potential biological, chemical, or immune effects associated with intrinsic properties of *P. regius* skin, including residual microbiota and biologically active components, we verified this by using ground skin samples and measuring bacterial growth in their presence over time. The samples were sterilized by sequential rinsing in ethanol followed by UV–C sterilization (30 min). Sterile skin fragments were subsequently ground into powder and added to culture medium (final concentration 1 g l^–1^) to assess residual activity. No increase in absorbance (turbidity) was observed spectrophotometrically in the enriched medium throughout the entire 24-h cultivation period. Bacterial cultures incubated in this medium (24 h, 37 °C) exhibited growth kinetics with key parameters indistinguishable from control cultures (bacterial suspension in nonenriched medium). These results confirmed that the treated skin samples were sterile and devoid of antimicrobial activity, thereby ensuring that subsequent experiments reflected the influence of surface topography alone (Figure 3).

**Figure 3:**
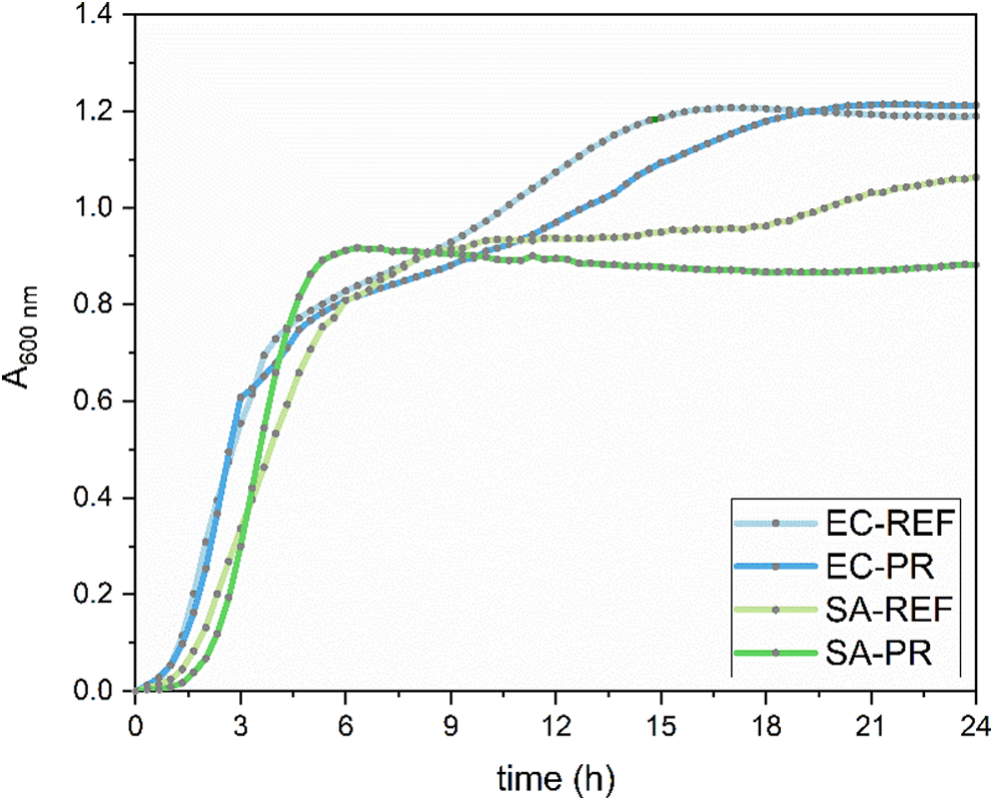
Growth curves of *E. coli* and *S. aureus* cultivated separately (EC-REF, SA-REF) and in medium enriched with a component from ground *P. regius* skin (EC-PR, SA-PR) monitored for 24 h.

Having verified the sterility of treated skin sheds and the absence of residual antimicrobial activity of its components, we next evaluated the effect of surface topography on bacterial adhesion and biofilm formation. A key methodological challenge was the spatial heterogeneity of the skin sheds: the sharp, spike-like protrusions of interest occur only on one face of each dorsal scale, leaving a substantial amount of nonstructured regions (interscale epidermis, underside of skin shed) that, if exposed, would permit conventional adhesion and thereby mask any topography-dependent effects. To remove this confound, we mapped the distribution of protrusion-rich regions and constructed a scale-pair model (Figure 4) that maximizes the area of exposed microprotrusions while minimizing unstructured surface exposure.

**Figure 4:**
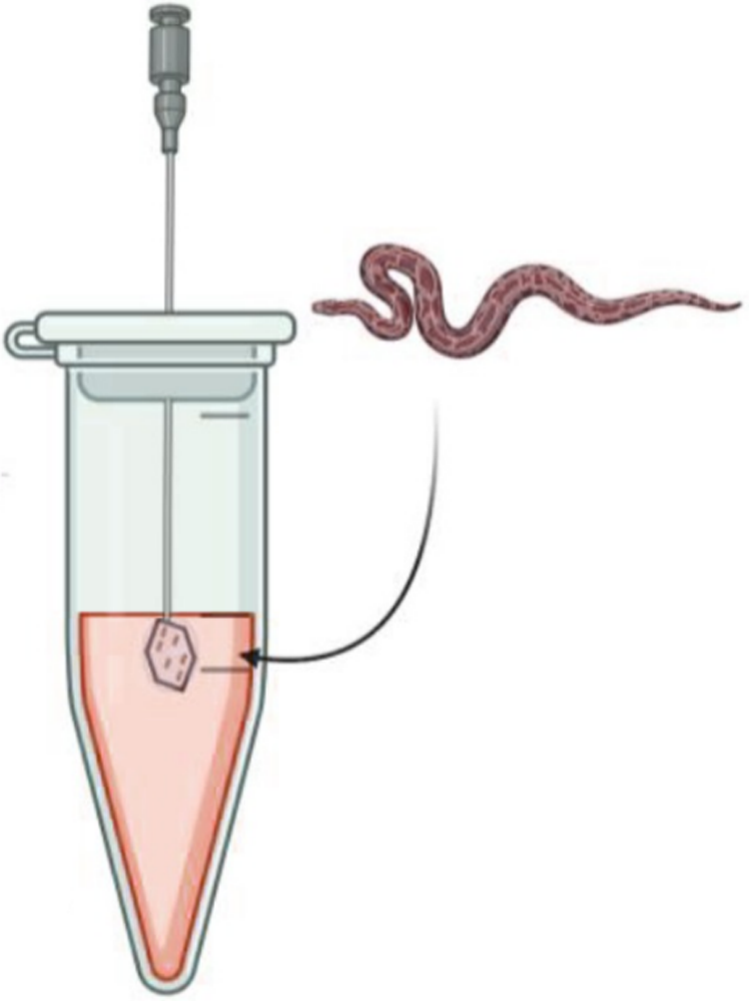
Scale-pair model design: two scales are glued together using acetate silicone and attached to a needle.

Scale-pair models were produced by bonding two selected shed scales back-to-back with biologically inert acetate silicone and mounting the assembly on the tip of a sterile stainless-steel needle secured in the cap of a microcentrifuge tube. This configuration presents an exposed surface area approximately equal to the sum area of the two scales and concentrates the spike-bearing topography across the whole model surface. Prepared assemblies were incubated as described above for 48 h to capture mature biofilm development based on prior kinetic data (Figure S3).

Following incubation, samples were processed and adherent cells quantified as CFU/cm^2^ (Figure 5), and representative specimens were imaged by SEM to document cell distribution, microcolony morphology, and the spatial relationship between bacterial adhesion/biofilm formation and scale topography (Figure 6). This experimental design isolates the effect of surface morphology by presenting a homogeneous field of sharp protrusions while controlling for chemical and material-related confounders.

**Figure 5:**
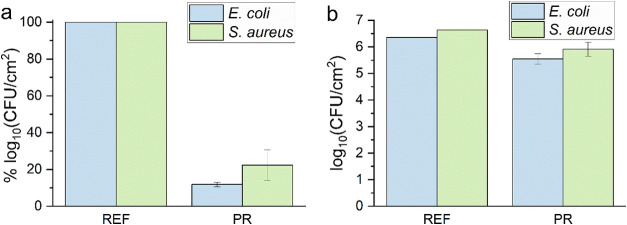
Biofilm formation (48 h) by *E. coli* and *S. aureus* on reference polystyrene surface (REF) and *P. regius* scale-pair model (PR) quantified using percentual evaluation (a) and CFU enumeration (b).

**Figure 6:**
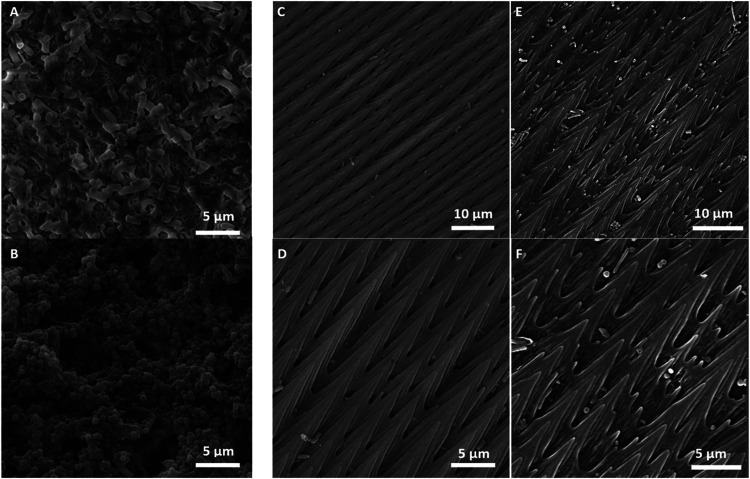
Bacterial biofilm formation (48 h) on reference polystyrene surface (A) *E. coli*, (B) *S. aureus*, and *P. regius* scale-pair model: (C, D) *E. coli*, (E, F) *S. aureus* visualized using SEM.

Scale-pair models with sharp microprotrusions produced striking and reproducible reductions in bacterial adhesion relative to smooth polystyrene controls: *E. coli* adhesion was reduced on average by 88%, and *S. aureus* adhesion by 78% (Figure 5). These reductions were consistent across replicates and were confirmed by statistical analysis (*t* test with Tukey’s post hoc test). Although the relative decreases in biofilm CFU/cm^2^ values on the scale-pair model may appear low, they represent a substantial reduction compared to controls. The absolute counts can be influenced by residual inhomogeneities in modelś surface topography, for example, at scale edges or at small regions of exposed acetate silicone or metal support—where unwanted adhesion may still occur. Complete elimination of such unstructured microdomains is challenging for natural materials, and no standardized assay currently exists to evaluate biofilm formation exclusively at microscopic loci of sharp protrusions. We therefore emphasize the importance of multimethod approaches when interpreting topography-driven effects to ensure reliable results. In this context, SEM appears to be an indispensable tool for surface analysis, which is consistent with previous studies in this field. SEM analysis confirmed sparse colonization of the spike-bearing scale surfaces by both species (Figure 6). Adherent cells were generally confined to sheltered interstices between protrusions and showed no hallmarks of mature biofilm architecture, with an apparent absence of extensive extracellular polymeric substance and of large, confluent microcolonies. A comparison with the control biofilms of both tested bacteria (Figure 6A,B) is particularly conclusive – during 48 h of cultivation, the bacteria in the control sample formed a mature, very dense biofilm. These results clearly demonstrate the antiadhesive properties of this part of the skin. Taken together, the quantitative CFU data and the SEM observations indicate that the spike-rich regions of the scales exert a pronounced antiadhesive effect that inhibits both initial attachment and subsequent biofilm maturation.

In principle, the inhibitory effect of snake scale surface topography can be attributed to a combination of mechanical and physical mechanisms: (i) a sufficiently dense array of regularly arranged, sharp protrusions substantially reduce the effective contact area available for bacterial adhesion, forcing cells into geometrically unstable adhesion with less firm bonds, thus preventing colonisation; (ii) the local nanoscale curvature of bacteria imposed mechanically by sharp edges may impose membrane deformation leading to localized stress of adhering cells. Exceeding the mechanical tolerance of bacterial cell envelopes can especially in Gram-negative species with thinner peptidoglycan layers, lead to reduced viability;^6,23^ (iii) the rugged geometry prevents or hinders EPS accumulation, thereby preventing the formation of the cohesive matrix necessary for microcolony coalescence and mature biofilm stability; (iv) complex surface topography, especially in combination with surface secretion of nonpolar lipids and other similar molecules, may contribute to reduced wettability of the skin surface, further limiting colonization. In our experiments, we neglect these phenomena because our scale-pair model, as well as treated shed skins, did not exhibit any degree of nonwettability under assay conditions; however, in living snakes, this phenomenon may further enhance the surface antimicrobial effect. All these effects are consistent with the predictions of the Derjaguin–Landau–Verwey–Overbeek colloidal adhesion theory (DLVO and its extensions), which emphasizes the importance of physicochemical factors (local energy landscapes, surface geometry, and mechanical forces) in determining bacterial adhesion.^24^

From an evolutionary standpoint, the antibacterial properties associated with *P. regius* scale topography likely confer a selective advantage in microbe-rich habitats. Most snake species occupy hot and humid environments rich in organic matter, where persistent contact with potential pathogenic microbial communities is unavoidable. Periodic ecdysis, in a way, already serves as an effective self-cleaning mechanism, mechanically removing accumulated contaminants, ectoparasites, and senescent or infected surface layers. The emergence of surface micro- and nanostructures capable of passively inhibiting microbial adhesion and biofilm formation would therefore provide an additional, complementary line of defense. Such a mechanism would reduce microbial burden between shedding events and lower infection risk without imposing a measurable metabolic cost, illustrating an elegant evolutionary strategy for maintaining integumentary hygiene through purely structural adaptation.

Based on our results and overall knowledge of *P. regius* scales, we believe biomimetic materials inspired by the observed surface topography could find application in medicine, where biofilm-resistant catheters, implant surfaces, and wound dressings are urgently needed for combating infections. In an industrial setting, analogous surfaces could mitigate biofouling of filter membranes, water treatment systems, and food processing equipment. Compared with chemical coatings that may deteriorate, leach active agents, or select for resistant strains, micro/nanotopography-based strategies offer inherent durability and reduced ecological impact.^25^ Emerging fabrication methods such as nanoimprint lithography, 3D laser structuring, and electrospinning enable scalable production of surfaces with controlled micro- and nanofeatures.^26,27^ By improving and directly translating design parameters observed on snake scale—feature size, spacing, sharpness, and anisotropy—synthetic analogues tailored for specific environments could be manufactured.

Moreover, this study opens avenues for integrating biology and materials science in a two-way dialogue. Just as materials engineers can learn from snake skin, biologists can revisit questions about the ecological significance of integumentary structures in reptiles. Comparative studies across snake taxa, habitats, and behaviors could reveal correlations between surface topography and its biological function, deepening understanding of how evolution shapes surfaces at the micro- and nanoscale to meet diverse ecological challenges.

In conclusion, our study demonstrates that the scales of *P. regius* skin sheds possess regular, sharp microstructures that inhibit bacterial adhesion and biofilm development by over 80%. This inhibition arises from physical surface topography, not biochemical antimicrobial activity. The results confirm snake scales as an overlooked model for natural antifouling strategies, and present new opportunities for biomimetic material design.

## Supplementary Material



## Notes

Figure S1: SEM analysis of sharp protrusions; Figure S2: FTIR spectra; detailed FTIR characterization; Figure S3: Biofilm formation by *E. coli* and *S. aureus* over a period of 48 h (PDF)
