# Supplementary material for: Microarchitecture of Python regius Scale Surface: A Natural Strategy for Bacterial Adhesion Prevention
Source: ACS Omega. 2026 Mar 11;11(11):18036–43. doi: 10.1021/acsomega.5c12739 (PMC13019405; doi:10.1021/acsomega.5c12739)
Supplement: Supplementary file 1 [file ao5c12739_si_001.pdf]

Microarchitecture of *Python regius* Scale Surface: A Natural Strategy for Bacterial Adhesion Prevention

Vaclav Peroutka[1*], Katerina Navratilova[1], Vera Jencova[2], Jana Jiresova[3], Jana Mullerova[2], Simona Lencova[1]

[1] Department of Biochemistry and Microbiology, University of Chemistry and Technology, Prague, Czech Republic

[2] Department of Chemistry, Faculty of Science, Humanities and Education, Technical University of Liberec, Liberec, Czech Republic

[3] Department of Physics and Measurements, University of Chemistry and Technology, Prague, Czech Republic

[*]Corresponding author: Vaclav Peroutka, peroutkv@vscht.cz

SUPPORTING INFORMATION

1. SEM analysis of *P. regius* skin patterns

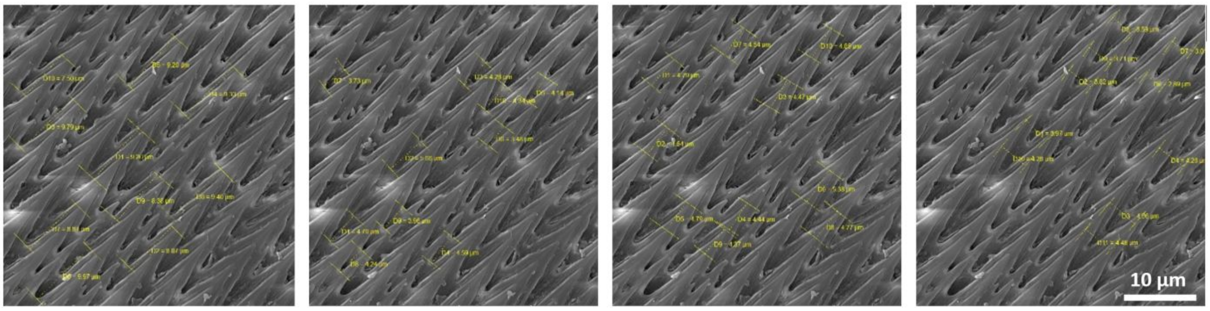

Figure S1: Sharp protrusions detected on *P. regius* skin, characterized in terms of their size.

2. FTIR

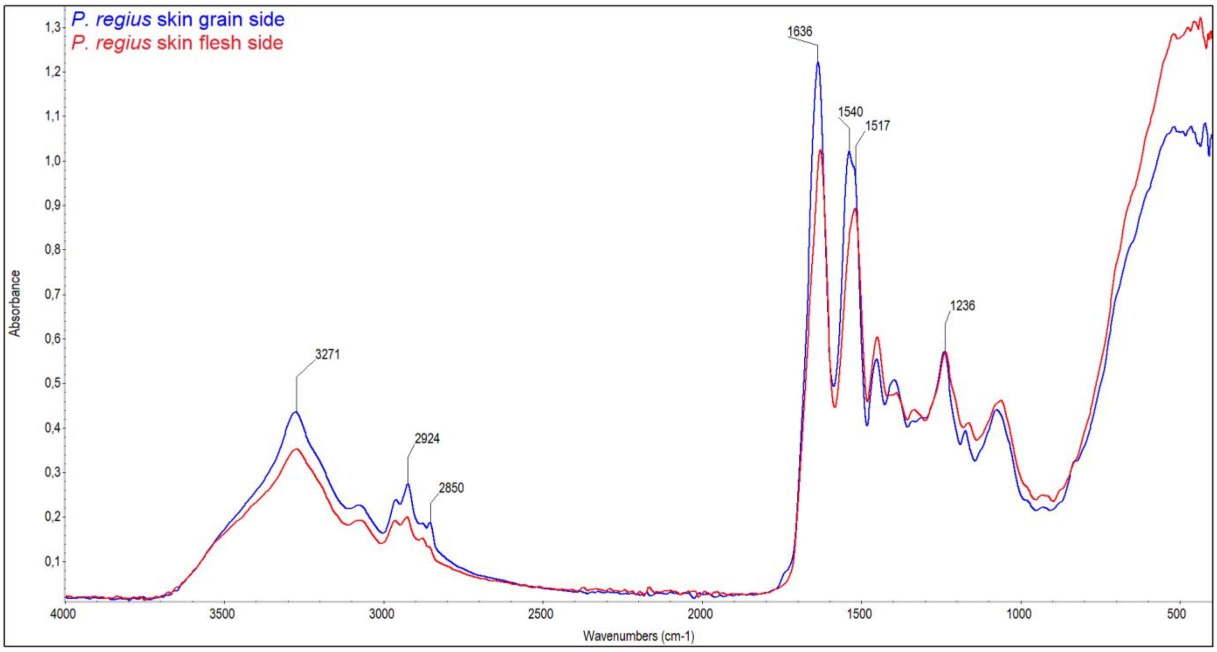

Figure S2: The obtained spectra were compared to the same intensity of the 1242 cm^{-1} band.

The grain side is identified as a keratin-rich, highly organized surface (epidermis), while the flesh side is identified as a collagen-based connective tissue (dermis) with a relatively higher lipid content. The differences visible from obtained FTIR spectra between the flesh and the grain side are:

1) In the intensity of N-H stretching vibration (amideA) of the peptide bonds in region ~3300 cm^{-1}. The broadening of the peak is due to extensive hydrogen bonding within the protein structure, which is characteristic of hydrated tissue samples[1] and more intense for the grain side of the sample.

2) In the intensity of C-H stretching in region 2850–2950 cm^{-1} (a doublet of peaks corresponding to asymmetric (~2920 cm^{-1}) and symmetric (~2850 cm^{-1}) stretching of $-CH_2-$ groups). These bands are primary markers for lipids in biological tissues.[2] The flesh side exhibits slightly more pronounced peaks in this region relative to the protein bands compared to the grain side. This is consistent with the histological structure of the dermis, which naturally contains higher residual subcutaneous fat content than the keratinized epidermis.

3) In the intensity of the most prominent peak in the spectrum (region ~1650 cm^{-1}) which corresponds to the C=O stretching vibration of the peptide backbone (amideII). This band is the most

sensitive marker for protein secondary structure.[1, 3] Grain side shows higher absorbance intensity. This is likely due to the high concentration of beta-sheet structures (typical of keratin) and a higly organised surface. Flesh side is composed mainly of collagen (triple helix structure), the peak position suggests a mixture of ordered and disordered structures [1].

4) In the intensity of the amideII peak (region 1540 + 1517 cm^{-1}), which corresponds to C-N and N-H bond vibrations, a distinct peak adjacent to amideI. The presence of both amideI and II is the confirmation of the polypeptide nature of the sample.

5) Differences in the region 1000–1300 cm^{-1} which is complex bands including amideIII (1240–1280 cm^{-1}) and C-O/C-O-C vibrations (1000–1100 cm^{-1}). This region (also called fingerprint region) is crucial for distinguishing specific protein types [1]. The flesh side displays spectral features consistent with type I collagen, which often shows specific absorption patterns in the 1000–1100 cm^{-1} range due to carbohydrate moieties (glycosaminoglycans) in the extracellular matrix**.** The grain side features are sharper, corresponding to the rigid, cross-linked structure of keratin.

3. Dynamic of bacterial biofilm formation

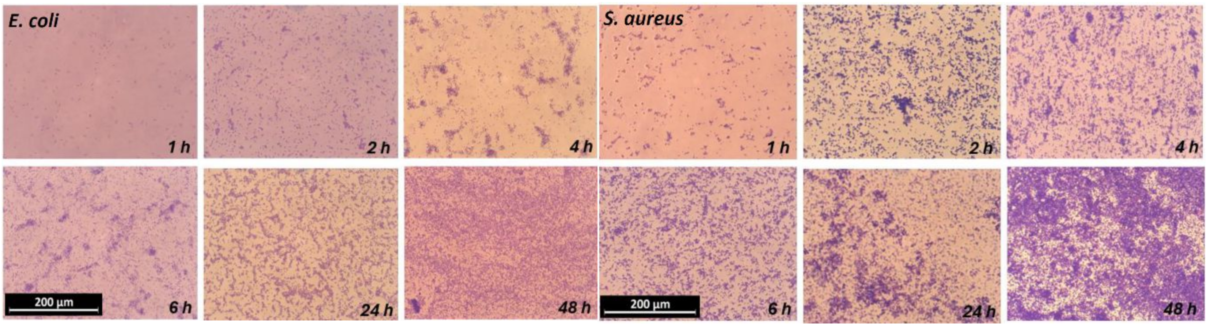

Figure S3: Biofilm formation by *E. coli* (on the left) and *S. aureus* (on the right) over a period of 48 hours; qualitative determination by staining the biofilm with crystal violet (the biofilm is stained purple).

References:

(1) Barth, A. Infrared spectroscopy of proteins. *Biochimica et Biophysica Acta (BBA) - Bioenergetics* **2007**, *1767* (9), 1073-1101. DOI: https://doi.org/10.1016/j.bbabio.2007.06.004.
(2) Baker, M. J.; Trevisan, J.; Bassan, P.; Bhargava, R.; Butler, H. J.; Dorling, K. M.; Fielden, P. R.; Fogarty, S. W.; Fullwood, N. J.; Heys, K. A.; et al. Using Fourier transform IR spectroscopy to analyze biological materials. *Nature Protocols* **2014**, *9* (8), 1771-1791. DOI: 10.1038/nprot.2014.110.
(3) Kong, J.; Yu, S. Fourier Transform Infrared Spectroscopic Analysis of Protein Secondary Structures. *Acta Biochimica et Biophysica Sinica* **2007**, *39* (8), 549-559. DOI: https://doi.org/10.1111/j.1745-7270.2007.00320.x (acccessed 2025/12/04).
